# Human-to-Human Position Estimation System Using RSSI in Outdoor Environment

**DOI:** 10.3390/s22197621

**Published:** 2022-10-08

**Authors:** Takashi Yamamoto, Tomoyuki Yamaguchi

**Affiliations:** 1 Master’s Programs in Intelligent and Mechanical Interaction Systems, University of Tsukuba, Tsukuba 305-8573, Japan; 2 Faculty of Engineering, Information and Systems, University of Tsukuba, Tsukuba 305-8573, Japan

**Keywords:** received signal strength indication, outdoor system, position estimation

## Abstract

Methods to prevent collisions between people to avoid traffic accidents are receiving significant attention. To measure the position in the non-line-of-sight (NLOS) area, which cannot be directly visually recognized, position-measuring methods use wireless-communication-type GPS and propagation characteristics of radio signals, such as received signal strength indication (RSSI). However, conventional position estimation methods using RSSI require multiple receivers, which decreases the position estimation accuracy, owing to the presence of surrounding buildings. This study proposes a system to solve this challenge using a receiver and position estimation method based on RSSI MAP simulation and particle filter. Moreover, this study utilizes BLE peripheral/central functions capable of advertising as the transmitter/receiver. By using the advertising radio waves, our method provides a framework for estimating the position of unspecified transmitters. The effectiveness of the proposed system is evaluated in this study through simulations and experiments in actual environments. We obtained an error average of the distance to be 1.6 m from the simulations, which shows the precision of the proposed method. In the actual environment, the proposed method showed an error average of the distance to be 3.3 m. Furthermore, we evaluated the accuracy of the proposed method when both the transmitter and receiver are in motion, which can be considered as a moving person in the outdoor NLOS area. The result shows an error of 4.5 m. Consequently, we concluded that the accuracy was comparable when the transmitter is stationary and when it is moving. Compared with conventional path loss, the model can measure distances of 3 m to 10 m, whereas the proposed method can estimate the “position” with the same accuracy in an outdoor environment. In addition, it can be expected to be used as a collision avoidance system that confirms the presence of strangers in the NLOS area.

## 1. Introduction

Collisions among people (including drivers and operators) in the outdoors occur when they are unable to determine each other’s distance or position. While conventional camera-type sensor systems record the information of objects that are visible directly as data on a map, the measurement data of non-line-of-sight (NLOS) areas, such as the corners of intersections, which cannot be seen directly, is difficult [1].

Therefore, two position measurement methods, which use wireless GPS [2] and propagation characteristics of radio signals [3], were developed for measuring the position in NLOS areas. The former method uses GPS to measure the position of people (receiver and transmitter) by trilateration depending on the communication time between the satellite and each device, and the position of each device is shared through a common server. This enables position measurements in areas, even NLOS areas, where communication with the satellite is possible. However, in this method, limitations such as the need to connect to a common server, privacy concerns, and inaccuracy of the position measurements due to the surrounding buildings, persist.

Various types of measurement methods employed the prorogation characteristics of radio signals, such as angle of arrival (AOA), time of arrival/time difference of arrival (TOA/TDOA), and a radio signal strength indicator (RSSI). Similar to the GPS method, these methods exhibit position measurement accuracy degradation, owing to the surrounding buildings. Nonetheless, the receiver can measure the transmitter’s position without a common server between them using the Bluetooth and Wi-Fi as radio signals. Therefore, this method began receiving wide attention in recent years.

Compared to GPS-based methods, RSSI-based measurements do not require a connection to a common server and can be anonymously used without privacy issues. While AOA- and TOA/TDOA-based methods require an array antenna to calculate the AOA and time synchronization at the receiver, respectively, RSSI can be acquired using basic communication devices, such as smartphones, and does not require special equipment. Therefore, the RSSI measurement method is relatively easy to implement. However, a common problem with wireless position measurement methods is that the accuracy of the position measurement decreases when obstacles, such as building structures, are present in the vicinity [4].

**Figure 1:**
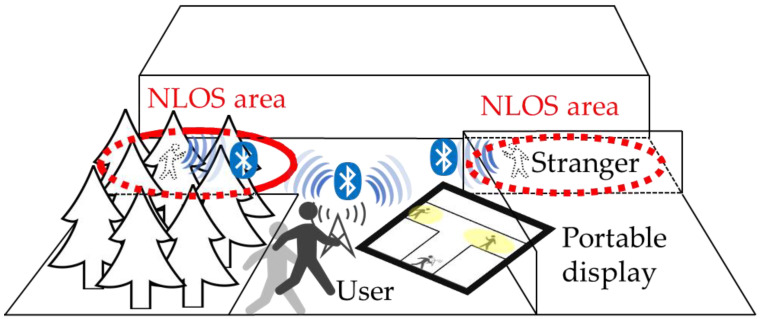
Conceptual diagram for human-to-human position estimation system using RSSI in an outdoor environment (NLOS area). User is the receiver and has a portable display map that estimates the location of surrounding strangers; transmitter, from RSSI values.

Therefore, this study proposes a novel method to measure the position of people to avoid collisions at outdoor intersections. The proposed method comprises a transmitter, which is a smartphone in this case, the Bluetooth RSSI of which is acquired (scanned) by the measurement device of the receiver, as shown in Figure 1. Conventional RSSI-based position measurement methods are based on trilateration and use multiple receivers with multiple receiving points. The receiver used in this study moves to acquire multiple RSSIs from the transmitter to achieve the same trilateration as the conventional methods using a single receiver. Considering the inaccuracy due to the surrounding buildings, a fingerprint model is developed in real time based on the map information, which includes the positions of the buildings and receiver, and an RSSI MAP is calculated for each measurement point of the receiver through simulation. Based on the relationship between the simulated and measured values, the position of the transmitter is determined using a particle filter. Simulation experiments and real-world measurement experiments are conducted to demonstrate the effectiveness of the proposed method. As Bluetooth is a commonly used technology available in smartphones, we believe that this method can be used as a cost-effective collision prevention strategy [4,5]. Thus, by estimating the position of strangers in the NLOS area, users (children and women) can prevent collisions and move on roads while avoiding strangers (avoid crime). This research can especially be used on the way home in the dark or in the suburbs as a safety application.

The remainder of this paper is organized as follows: Section 2 describes related research. Section 3 presents the proposed method that includes the RSSI MAP creation method and position measurement method based on the particle filter. Section 4 details the simulation experiments. Section 5 presents the experiment results in an outdoor environment with transmitter in stationary and moving states, as well as includes a discussion on the results. Section 6 presents the conclusions.

## 2. Related Works

There are two methods for measuring the transmitter position using RSSI: the path loss model [4,5] and fingerprint model [6,7]. The path loss model approximates the relationship between the transmission/reception distance and RSSI with a logarithmic decay formula and calculates the distance from the acquired RSSI. Bullmann et al. [4] measured the position by trilateration using a RSSI of radio waves received by a receiver from a moving transmitter (smartphone) in a hallway, with each room comprising a 2.4 GHz Wi-Fi receiver, and compared the results with fine timing measurement (FTM). Results show that the values of RSSI and FTM were 4.47 m and 3.52 m, respectively. Huang et al. [5] conducted an experiment in an indoor corridor where modules transmitting BLE radio waves were installed at every 3 m interval, and a person moved across the corridor with an android device that received the BLE radio waves. They proposed a position measurement method that utilizes the RSSI of radio waves received by a receiver from a transmitter with dead reckoning, using an accelerometer and a magnetic sensor (the accuracy of position measurement was 3–10 m).

Conversely, in the fingerprint model, multiple receivers are installed in the measurement area. The RSSI at each measurement point is obtained in advance, and the RSSI MAP of the entire measurement area is created as a database. Based on this RSSI MAP, the position of the transmitter is measured by matching with the RSSI acquired during system operation. Yasumine et al. [6] proposed a Wi-Fi RSSI fingerprinting method for self-positioning in shopping malls. As a result, this method improves accuracy by selecting an access point for calculation from among multiple access points (receivers) for each measurement location. Jiang et al. [7] proposed a BLE RSSI fingerprinting method for self-position measurement in a 5 × 8 m indoor area using an autoencoder (AE) or principal component analysis (PCA) to effectively reduce data from multiple access points.

Therefore, the path loss model is computationally inexpensive, as the relationship between the radio transmission/reception distance and RSSI can be expressed numerically. However, in environments where there are effects of radio wave reflections, such as changes in the surrounding environment and obstacles, the RSSI that includes the effects of reflections is approximated by a logarithmic decay equation with respect to the radio wave transmission/reception distance, causing a large error. In contrast, the fingerprint model has a smaller error than the path loss model, considering it measures the RSSI including the effect of reflection in the RSSI MAP measured in advance, even in an environment with the effect of radio wave reflection. However, the fingerprint model requires an RSSI MAP in advance, which is expensive to implement and has a limited measurement range.

Therefore, these methods can mostly be used for indoor GPS, which requires the preparation of multiple access points. Furthermore, it requires a connection to a common server, which increases privacy issues. Therefore, it is difficult to apply this technology for measuring the position between people outdoors, which is the subject of this study.

Conversely, RSSI-based methods are not studied extensively in outdoor environments, owing to the availability of GPS measurements, and hence, are used in limited locations or indoor applications. Llorca et al. [8] used the stereo measurement of position using the path loss model of RSSI to measure the position of a person using a roadside machine at a pedestrian crossing. Although this was an outdoor example, it is limited to the range of a pedestrian crossing. Furthermore, as a countermeasure against infection in the current coronavirus epidemic, the RSSI method was used in Japan for COCOA, an application that allows anonymous confirmation of contact with Covid-positive persons. When smartphones are near each other—approximately 1 m or less—for more than 15 min, it is detected as “contact” [9]. Leith and Farrell [10] studied the accuracy of distance measurement using the path loss model of RSSI in several scenarios in order to measure the distance between people to prevent coronavirus transmission. However, these methods require application registration or are insufficient for position measurement, considering they only measure the distance between people and not the position of one person with respect to another.

Therefore, the method of sharing the position information among users using GPS/indoor to avoid contact between people in outdoor environments is considered ineffective, owing to privacy issues, use of common servers, and limited application scope.

To solve these problems, this study aims to develop a system that can be used within the range of the user’s perspective, instead of the entire environment, to prevent outdoor accidents between a person (receiver/user) and a stranger (transmitter/stranger) and minimize the information received from others. The proposed system utilizes BLE peripheral functions capable of advertising as the transmitter. The receiver BLE device can scan the BLE central functions. By using the advertising radio waves, our method provides a framework for estimating the position of unspecified transmitters (stranger).

Furthermore, considering only a person (receiver and user) acquires information about his/her surroundings, this method enables measurement using a single receiver and computation of fingerprinting with a limited computational range in real time, and responds to changes in the surrounding environment.

## 3. Proposed Method

### 3.1. Overview

**Figure 2:**
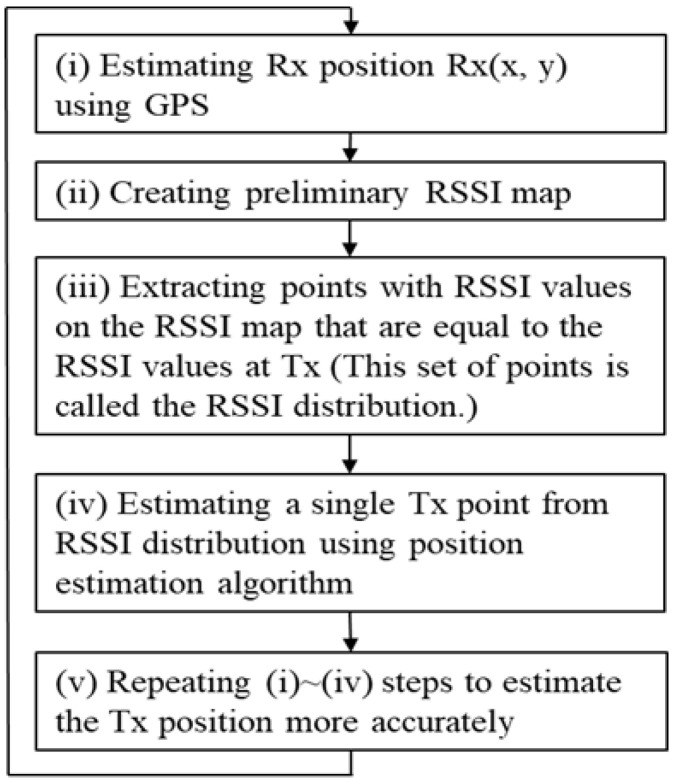
Flowchart of the proposed method.

The proposed method is a novel method that measures the position of strangers (radio wave transmitter, smartphone: Tx) around the user (radio wave receiver: Rx) (20 m × 20 m) based on the RSSI from the BLE-equipped smartphone to avoid collisions between people at an outdoor intersection. Figure 2 shows the flow of the proposed method.

**Figure 3:**
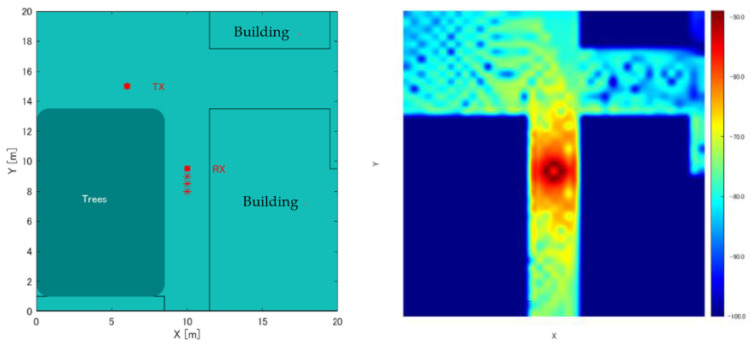
Examples of RSSI MAP generation. (**left**) 2D model including surrounding building information. (**right**) RSSIMAP based on Rx (colors represent strength of RSSI value).

(i) First, the position of Rx; Rx(s) = (rx(s), ry(s)) is obtained from the GPS. (ii) Then, a 20 m × 20 m environmental map information centered on Rx(s) is obtained from 3D environmental geometry information of urban areas, which recently became open source, where s is the number of iterations [11]. A grid (measurement points) is determined at 0.5 m intervals in the generated environmental map. RSSI from each measurement point is simulated, and the RSSI MAP:RSSIsimulation(x,y) of the entire measurement area is created as a database. A detailed procedure for creating the RSSI MAP will be explained in Section 3.2. Here, the origin of the RSSI MAP coordinates is assumed to be at s = 0. Figure 3 shows the RSSI MAP. (iii) Furthermore, we compare RSSI:RSSItx(s) from Tx(s) = (tx(s), ty(s)), and RSSIsimulation(x,y) to determine the matching candidate positions. We assume k to be the number of candidates. (iv) Considering multiple CandidateTx(k) matching RSSItx(s) are detected in the previous step, a particle filter is used to derive Tx(s) from CandidateTx(k) in order to estimate a single location from multiple candidates. (v) Lastly, we move Rx, a single receiver, and update the estimated position of Tx(s) by repeating the entire process.

Herein, we describe the equipment used in this study. First, the transmitting and receiving antennas are assumed to be half-wavelength dipole antennas, which are omnidirectional in the plane direction, mounted vertically at a height of 1 m from the floor (assumed height for human use). A Google Pixel 4a (Google LLC, Mountain View, CA, USA), capable of advertising BLE peripheral functions, is used as the transmitter. The receiver BLE device is the LINBLE-Z1, which can scan the BLE central functions.

### 3.2. RSSI MAP Creation by Simulation

The RSSI MAP calculated in a 3D model that was constructed to imitate a real environment uses the imaging method of the ray tracing method, which calculates the propagation of radio waves as rays of light [12]. Other radio propagation simulation methods include the FDTD method [13] and the ray launching method of the ray tracing method [14]. While the FDTD method is highly accurate, the amount of calculation increases as the propagation range increases. Conversely, while the ray launching method is computationally inexpensive, it has a low estimation accuracy. Furthermore, compared to these methods, the imaging method of the ray tracing method requires less computation time and can perform simulations with a certain level of accuracy if the surrounding environmental geometry is known and the conditions for the number of radio wave reflections are small. Therefore, this study uses the imaging method of the ray tracing method to perform a preliminary RSSI simulation within a 3D model constructed to imitate a real environment. Herein, the positions x and y, at which RSSI is calculated are grid points positioned every 0.5 m. Buildings are excluded for efficiency. As for the building material, considering an outdoor environment is used, the relative permittivity is set to 7, assuming that all floors and walls are dry concrete. To simplify the calculations, all building heights are assumed to be 20 m; the buildings are implemented in a planar geometry. With these conditions, the specific procedures used in this study are described below.

RSSI in RSSI MAP can be calculated based on free propagating and reflected waves. The free propagating wave *RF*(*x*, *y*) is the radio wave that reaches *Rx*(*x*, *y*) from *Tx*(*x*, *y*) without reflection, and is calculated as:

(1) $$RF\left(x,\;y\right)=\left(\cos\left(\frac{2\pi fD}{c}\right)-j\times \sin\left(\frac{2\pi fD}{c}\right)\right)\times 10^{\frac{10log10\left({\left(\frac{c}{4\pi fD}\right)}^{2}\right)+TP+Gt+Gr+RT\;\;}{10}}$$

where *Gt* [dBm] is the antenna gain of the transmitter, *Gr* [dBm] is the antenna gain of the receiver, and *RT* [dBm] is the attenuation value owing to wall transmission. Where *TP* [dBm] is the transmit power, which is RSSI when the receiver and transmitter are placed 1 m apart beforehand, *f* = $2.4\times 10^{9}$ [Hz] (frequency 2.4 GHz) is the frequency of radio waves, *c* = $3\times 10^{8}$ [m/s] is the speed of radio propagation, and $D=\sqrt{{\left(\mathrm{rx}\left(s\right)-\mathrm{tx}\left(s\right)\right)}^{2}+{\left(\mathrm{ry}\left(s\right)-\mathrm{ty}\left(s\right)\right)}^{2}}$ [m] is the distance between the receiver and the transmitter.

Next, for the reflected wave *RR* (*x*, *y*), the RSSI value of the radio wave (reflected wave) arriving from *Tx*(*x*, *y*) to *Rx*(*x*, *y*) reflected from the wall or floor, is calculated as:

(2) $$RR\left(x,\;y\right)=\overset{Ref}{\underset{i}{\sum }}\left(\cos\left(\frac{2\pi fD\left(i\right)}{c}\right)-j\times \sin\left(\frac{2\pi fD\left(i\right)}{c}\right)\right)\times 10^{\frac{10log10\left({\left(\frac{c}{4\pi fD\left(i\right)}\right)}^{2}\right)+TP+Gt\left(i\right)+Gr\left(i\right)+RT\left(i\right)+RD\left(i\right)\;\;}{10}}$$

where *i* denotes the number of reflected waves (reflected once at a wall or a floor and transmitted at other walls) that reach from the receiver to the transmitter, with the maximum number of reflections being one. Ref is the maximum value of *i*.

*RD*(*i*) [dBm] is the attenuation owing to wall reflection, *D*(*i*) [m] is the distance between the receiver and transmitter, and RT and RD are calculated from the incident angle and relative permittivity using Fresnel’s formula, respectively. TP is the transmit power [dBm], which is same as the RSSI value when the receiver and transmitter are placed 1 m apart beforehand.

The final RSSI MAP; *RSSIsimulation*(*x*, *y*) is calculated as:

(3) $$RSSIsimulation\left(x,y\right)=10\times \log_{10}\left|RF\left(x,\;y\right)+RR\left(x,\;y\right)\right|\;\left[dBm\right]$$

As described above, Equations (1) and (2) can be calculated for each grid, and *RSSIsimulation*(*x*,*y*) can be simulated as in Equation (3).

### 3.3. Position Measurement Method Based on Particle Filter

**Figure 4:**
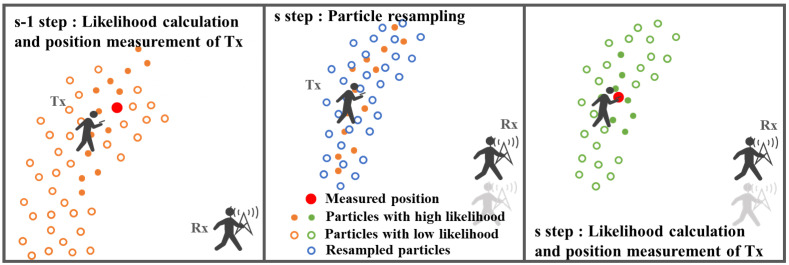
Flow of position estimation by particle filter. (**left**) likelihood calculation and position measurement in previous step. (**center**) resampling of particles when the Rx is moved in current step. (**right**) likelihood calculation and position measurement in current step.

Assuming the timing of the position measurement is represented by steps s = 0, 1, 2, …, the following can be performed—A. particle sampling, B. likelihood estimation, and C. position estimation; at every step s = 0, 1, 2, … in the position estimation phase (Figure 2 (iv)). The outline of the position estimation using the particle filter in the proposed method is shown in Figure 4, and the details are as follows:

A. Particle sampling

When s = 0, in the flow in Section 3.1, s = 0, i.e., only at the beginning; random scatter (sampling) particles (not a grid) in an M × M area around Rx (0) with a density count of 1 particle/m^2^, and their position is P(n) = (px(n), py(n)). Then, RSSIP(n) is the RSSI of the particle. The initial RSSIP(n) is the value of RSSIsimulation(x,y) closest to P (n); n is the number of particles. The total number of particles present in the measurement area is N.

When s ≥ 1, the RSSIP(n) at s-1 is used to classify all particles into groups with high and low likelihood using the K value obtained below as the threshold.

(4) $$K=\frac{1}{\sigma \sqrt{2\pi }}\times e^{-\frac{1}{2}{\left(\frac{SE}{\sigma }\right)}^{2}}$$

where *SE* and *σ* are the standard error and standard deviation of *X*(*n*), respectively, and are expressed as:

(5) $$SE\;=\;\sqrt{\frac{1}{N\times \left(N-1\right)}\times \overset{N}{\underset{n}{\sum }}{\left(X\left(n\right)-Xu\right)}^{2}}\;\;:\;Standard\;error\;of\;X\left(n\right)$$

(6) $$\sigma \;=\;\sqrt{\frac{1}{\left(N-1\right)}\times \overset{N}{\underset{n}{\sum }}{\left(X\left(n\right)-Xu\right)}^{2}}\;\;:\;Standard\;deviation\;of\;X\left(n\right)$$

where *X*(*n*) and *Xu* are expressed as:

(7) X(n)=|RSSIP(n)−RSSItx(s)|

(8) $$Xu\;=\frac{1}{N}\times \overset{N}{\underset{n}{\sum }}X\left(n\right)$$

where *n* is the number of particles and *N* is the total number of particles present in the measurement area; s is the number of steps assumed for position measurement timing. *RSSIP*(*n*) is the RSSI of the *n*-th particle, *RSSItx*(*s*) is RSSI measured at *Tx*(*s*) = (tx(*s*), ty(*s*)). Then, particles with low likelihood are randomly scattered (resampling) around the particles with high likelihood. Here, the area of scattering should be within 2 m around the particle classified, considering the high likelihood score and the number of scattered pieces should be at a density of 1 piece/$m^{2}$.

B. Particle likelihood estimation

The difference between the *RSSIP*(*n*) and the measured *RSSITx*(*s*) is calculated, and the likelihood *L*(*n*) of each particle is calculated as:

(9) $$L\left(n\right)=\frac{1}{\sigma \sqrt{2\pi }}\times e^{-\frac{1}{2}{\left(\frac{X\left(n\right)}{\sigma }\right)}^{2}}$$

where *X*(*n*) and *σ* in are taken from Equations (6) and (7).

C. Position measurement

The general procedure for the particle filter position measurement is to estimate the position of Tx using a weighted average of the position coordinates of each particle, using the likelihood of each particle as weights based on *L*(*n*) in Equation (9).

**Figure 5:**
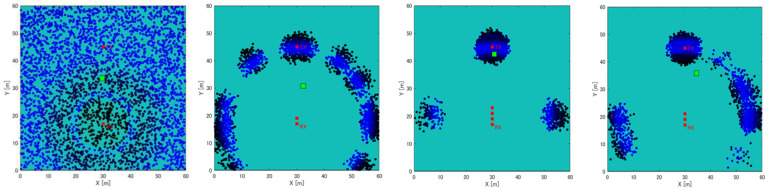
Distribution of the particles in wildness. The difference in distribution, depending on the number of steps. The green dot represents the estimated position of Tx.

**Figure 6:**
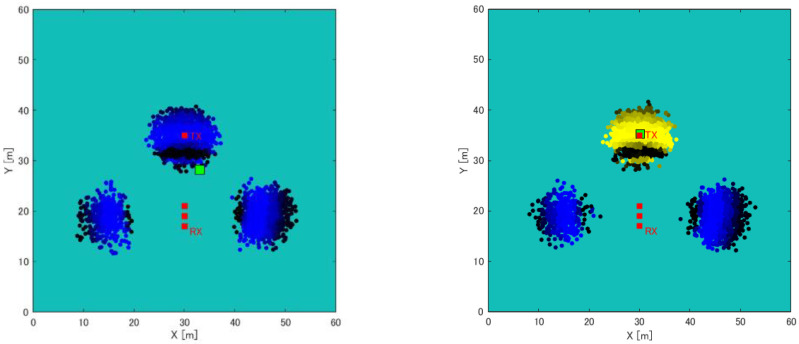
Position estimation results of measurement simulation using a particle filter. Left: weighted average; right: label method. The green dot represents the estimated position of Tx.

As shown in Figure 5, weighted averaging can be performed accurately when particles converge to a single location while the number of steps s increases. However, when particles are scattered (symmetrically distributed), the position measurement does not work well, as shown in Figure 6. Therefore, the particle labeling process in this study is performed after the likelihood estimation of B to measure the position. Furthermore, the labeling process enables accurate position measurement, even when particles are scattered, as shown in Figure 6. The following procedure is followed for particle labeling:

(i) Label the particle with the largest likelihood and the particles within 2 m.
(ii) Label particles within 2 m of the labeled particles.
(iii) Repeat step (ii) until labeling is no longer possible.

Then, for each labeled particle, a weighted average is calculated using the likelihood of each particle as weights, as shown in Equation 10 below, to measure the position *Tx*(*s*).

(10) $$Tx\left(s\right)=\overset{Q}{\underset{q}{\sum }}\frac{L\left(q\right)\times P\left(q\right)}{N}$$

where *q* is the number of labeled particles and *Q* is the total number of labeled particles.

## 4. Experiments Using Simulation

### 4.1. Simulation Experiment of Position Estimation Using Artificial Map

**Figure 7:**
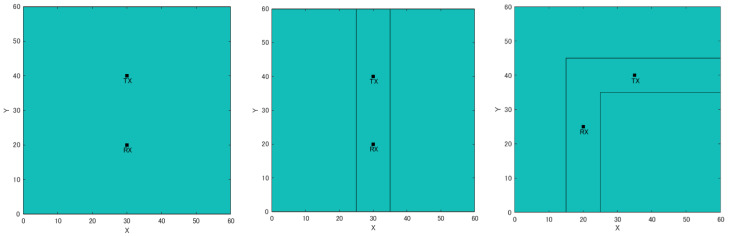
Artificial created maps (wilderness, straight road, and L-shaped road).

A simulation experiment was conducted to confirm the effectiveness of the proposed method using a single receiver and RSSIMAP. First, we prepared three different artificially created maps (wilderness, straight road, and an L-shaped road). In each map, the distance between Rx and Tx was changed by 1 m, from 5 m to 30 m. Additionally, the movement of a single receiver was compared when the number of steps was 3 and 5. The calculation of position estimation is evaluated for weighted average and clustering. Figure 7 shows artificially created maps (wilderness, straight roads, and L-shaped roads). Here, the width of each road is 10 m, and Rx and Tx are located in the middle of each road. Appropriate values for the number of particles, the range of particle scattering, and the number of moving steps of the receiver were determined as experimental parameters through preliminary experiments. Here, since the RSSI MAP is calculated with a resolution of 0.5 m intervals, it is conceivable to set the number of particles to 1 per 0.5 m^2^. However, as the number of particles increases, the calculation time increases, so we decided to scatter the number of particles at a rate of 1 per m^2^. Particles were scattered (resampled) in a 2 m range around particles with high likelihood. Here, the resampling range is based on the human walking speed, and we assumed that the walking speed is within 1 to 2 m/s, so the resampling range was set to 2 m.

For the computational cost, by limiting the resolution of the RSSI MAP, the number of particles scattered by the particle filter, and the range of position estimation, the time for one position estimation is set to 1 s. However, when the position estimation range is doubled, the RSSI MAP creation time and the number of particles increase, and the calculation time increases proportionally.

**Figure 8:**
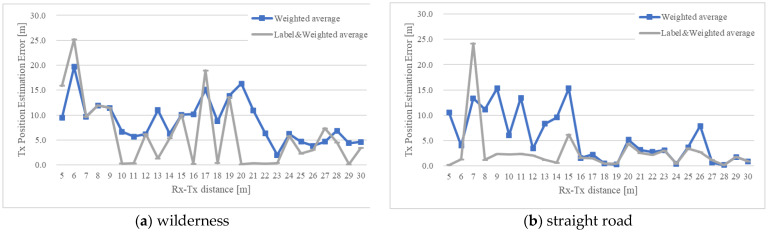

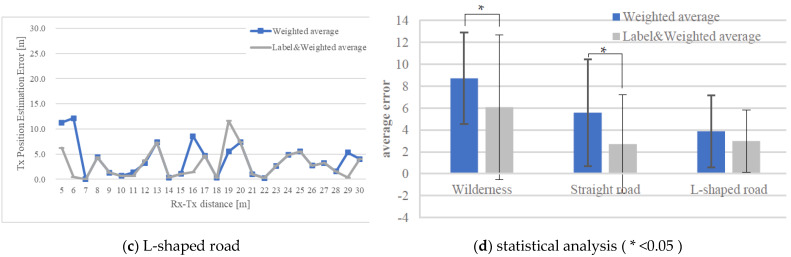
Position estimation results of the measurement simulation using the particle filter (three-step RSSI measurement). (**a**) wilderness; (**b**) straight road; (**c**); L-shaped road; (**d**) statistical analysis.

**Figure 9:**
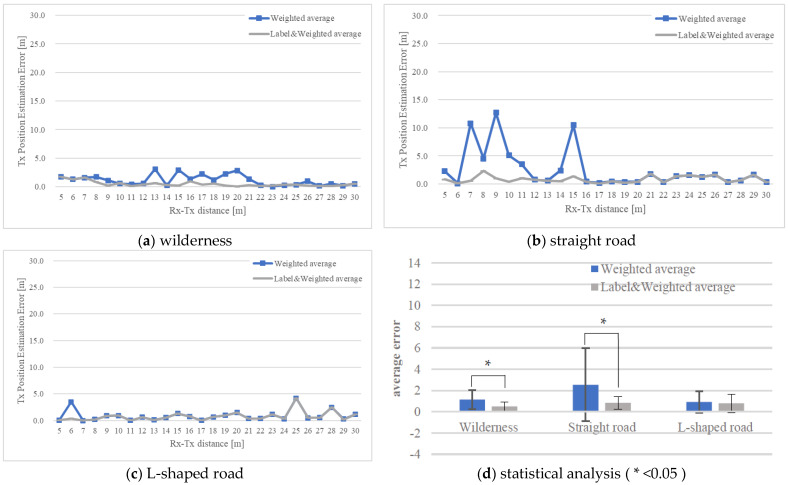
Position estimation results of the measurement simulation using the particle filter (five-step RSSI measurement). (**a**) wilderness; (**b**) straight road; (**c**); L-shaped road; (**d**) statistical analysis.

Figure 8 and Figure 9 show the position estimation accuracy in each map when the number of steps is 3 and 5, respectively. It can be seen that as the number of steps increases, the accuracy improves, considering the particle distribution converges as the number of steps increases. Next, the difference between the maps is noticeable in Figure 8. In the case of a symmetrical building arrangement, such as a wilderness or a straight road, particles with high likelihood are distributed symmetrically or in an anteroposterior manner. Therefore, as described in Section 3.3, if the number of steps is small and the particles are symmetrically distributed, the error will be large in the weighted average position estimation calculation. Conversely, in the case of the labeling method, if an incorrect label is selected (for example, when (a) Rx-Tx in Figure 8 is 6, the error may be large), the overall appropriate label is considered to be the final one. It was verified that if the labels were selected, the accuracy was improved compared with the selection of weighted average.

Moreover, Figure 8d and Figure 9d show the results of each average from (a) to (c) the weighted average, as well as label and weighted average. Then, the paired t-test technique (two-sided) was employed to verify the results. From the *t*-test for three steps in Figure 8d, we determined whether there is a statistically significant difference for wilderness t(25) = 2.555, *p* < 0.017 and straight road t(25) = 2.819, *p* < 0.009. From the t-test for five steps in Figure 9d, we determined whether there is a statistically significant difference for wilderness t(25) = 3.585, *p* < 0.001 and straight road t(25) = 2.550, *p* < 0.017. However, there were no significant difference for the L-shaped road of three steps (t(25) = 1.414, *p* < 0.170), and five steps (t(25) = 0.999, *p* < 0.328). From these results, it was confirmed that the use of label and weighted average has a higher accuracy than weighted average alone.

### 4.2. Simulation Experiment of Position Estimation Using a Map Based on the Real Environment

**Figure 10:**
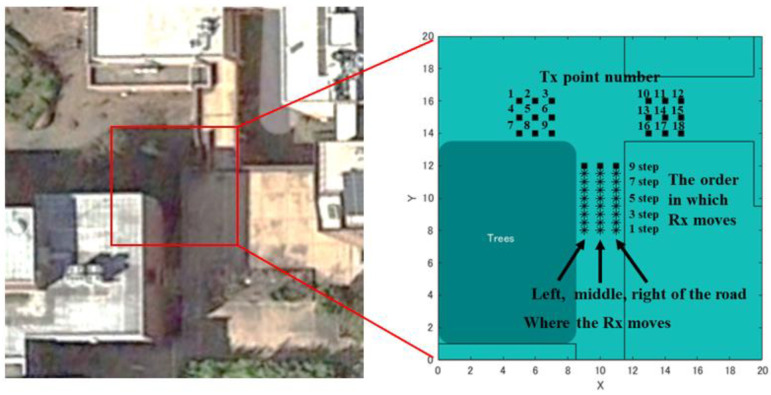
View of T-shaped road, and the setting of Rx movements and Tx positions.

**Figure 11:**
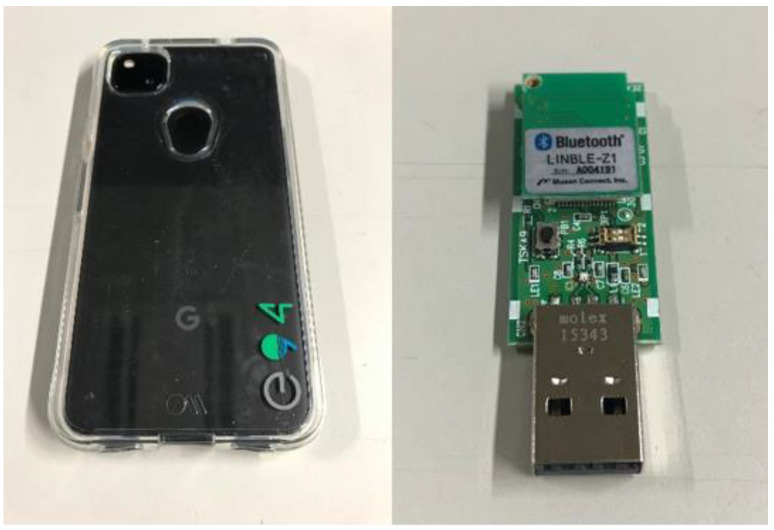
View of Pixel 4a for Tx and LINBLE-Z1 for Rx.

**Figure 12:**
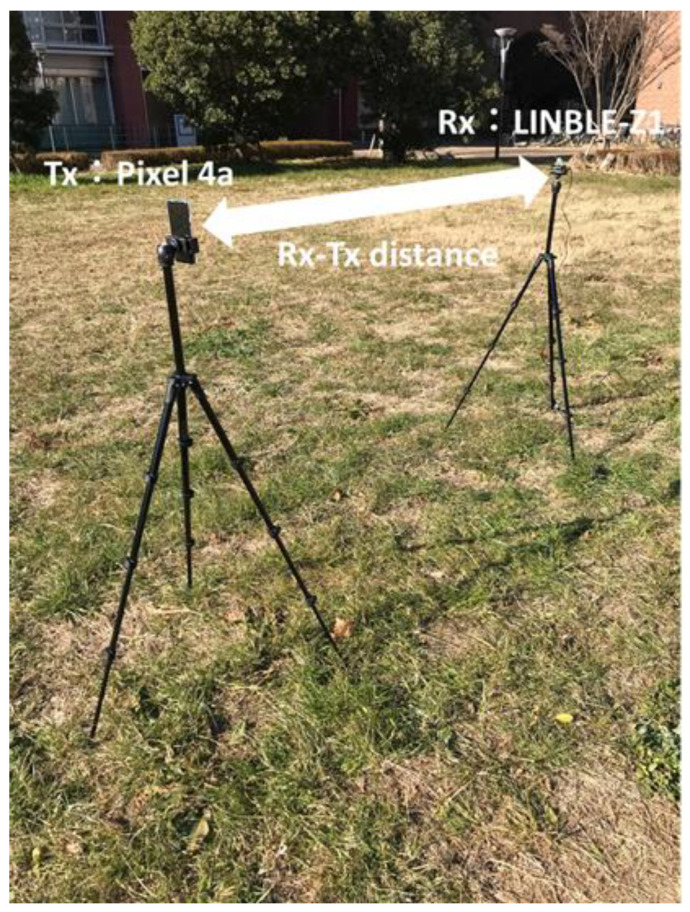
Appearance of RSSI measurement in the wilderness.

**Figure 13:**
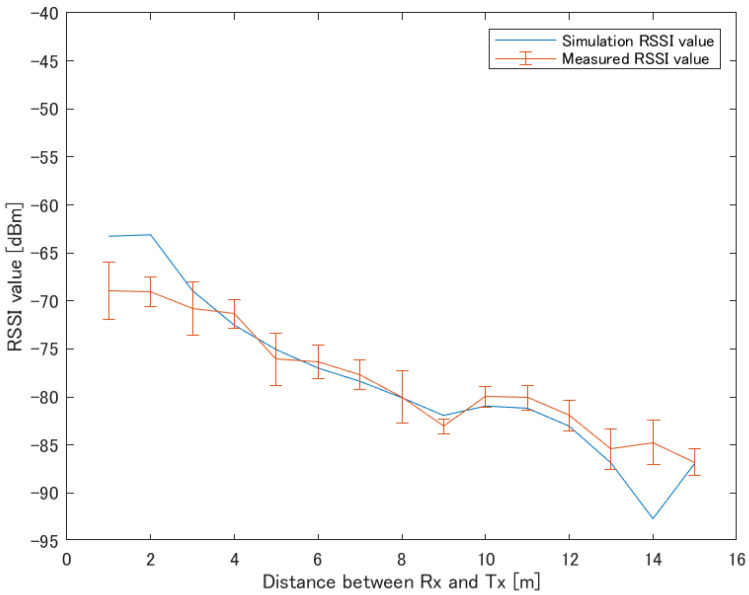
RSSI results with LINBLE-Z1 in the wilderness.

This study aims to construct a human-to-human position estimation system, and the accuracy with which the user estimates the positions of people around him is important. So, we investigated the differences in the positions of people in the real environment. On applying the proposed system outdoors, it is essential to consider both the relationship between the distance between Rx and Tx and the positional relationship between Rx and Tx. Therefore, we created a map based on the actual environment and verified the change in accuracy based on the positional relationship between the Rx movement path and Tx. We considered an actual environment, i.e., a T-shaped road, which is symmetric and 2D, as shown in Figure 10. This time, the 2D map was created manually. Figure 10 shows the position of the Rx and Tx used for evaluation. Rx changes its way of movement along the road, left, center, and right. The distance of Rx and Tx from the wall is 1.5 m, and the measurement is performed at 0.5 m intervals until the Rx and Tx are within the visible area (LOS) range. In addition, the positional relationship between Tx and Rx is assumed to be within the NLOS area. Next, the correct answer data are recorded to obtain the correct RSSI in the real environment. As shown in Figure 11, using the LINBLE-Z1 as the receiver and the smartphone as the transmitter, the output of the transmitter is fixed to the maximum output, and the LINBLE-Z1 scans the advertising radio waves from the transmitter. RSSI measurement is performed only by operation. However, the RSSI by simulation and the RSSI actually measured are affected by noise, as well as the effect of the building, and hence, require offset adjustment. Therefore, a receiver and a transmitter are installed in the wilderness, as shown in Figure 12. Then, RSSI measurement is performed 60 times per minute at each Rx-Tx distance. Furthermore, the offset adjustment is performed using the RSSI value obtained by averaging the measured values. Figure 13 shows the result. Therefore, the RSSI value in the simulation and the actual measurement are almost the same.

As an experimental procedure, while Tx is stationary at a specific point on the right or left side of the road with respect to the T-junction, Rx moves in one of three types (left, center, and right) directions to perform RSSI measurement at each point on the trajectory. A particle filter is applied using each RSSI value calculated in this simulation, and the position is estimated using the labeling method. The position estimation error for each Tx position and Rx movement is measured and evaluated by the average error of 3–9 steps.

**Figure 14:**
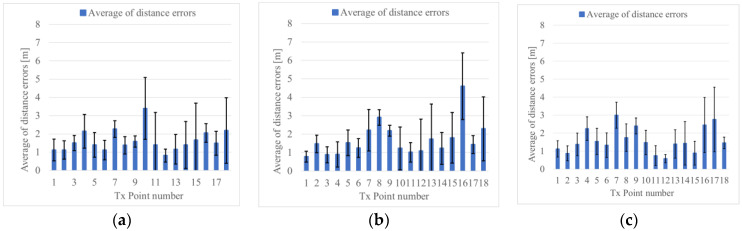
Position estimation results of measurement simulation in the T-shape road. (**a**) Rx moves to the left side of the road, (**b**) Rx moves to the middle of the road, (**c**) Rx moves to the right side of the road by the wall.

As a result of the position estimation simulation, when Rx moves on the left side, center, and right of the road, the error average of the distance is 1.6 m, 1.7 m, and 1.6 m, as shown in Figure 14a–c, respectively. The average error of the distance was 1.6 m, as a whole

The average error of the distance was 2 m or less. From Figure 14, it can be confirmed that when Tx points are near a corner, such as 7, 8, 9, 16, 17, and 18, the average error of the distance is 2 m or more. This can be attributed to the fact that when the position of Tx is close to the Rx; the error increases because appropriate clusters are not formed considering there are many same RSSI values.

## 5. Experiment in an Outdoor Environment

### 5.1. Actual Measurement Experiment with Tx Stationary State

Similar to the simulation experiment conducted in Section 4.2, the position estimation experiment is performed using the actually measured RSSI. As for the RSSI measurement method, a transmitter and a receiver are installed, as in the offset adjustment in the previous section, and the measurement is performed 10 times for 10 s at each point. Additionally, the position estimation of each Rx movement for each Tx point in nine steps was repeated three times, and the evaluation was performed using the value obtained by averaging the errors. Herein, the BLE radio waves observed in the measurement environment were all −90 dBm or less, except for that of Pixel 4a, with minimum radio wave interference. The experiment was conducted on a day when the weather was fine and the wind speed was less than 5 m.

**Figure 15:**
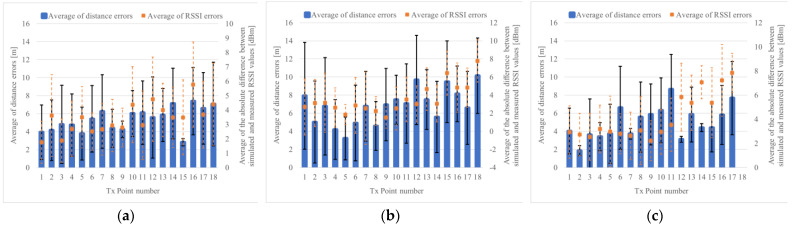
Position estimation results of actual measurement in T-shape road. (**a**) Rx moves to the left side, (**b**) Rx moves to the middle, (**c**) Rx moves to the right side.

**Figure 16:**
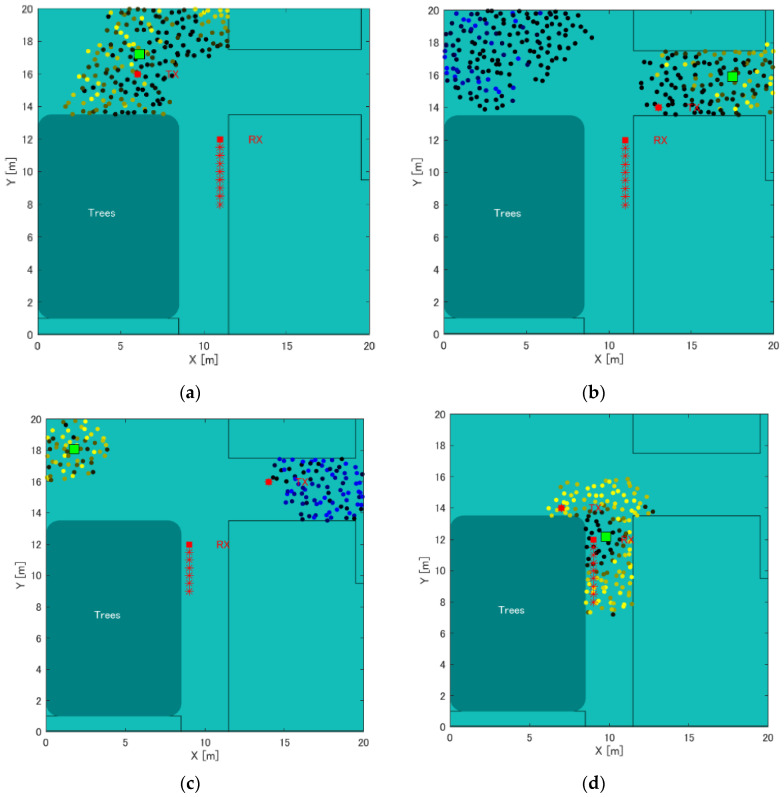
Comparison of the error average of distances in the position estimation result on T-shape road. (**a**) Tx on the left (Tx Point number 2), when Rx moves to the right side of the road by the wall, (**b**) Tx on the right (Tx Point number 16), when Rx moves to the left side of the road, (**c**) Tx on the right (Tx Point number 10), when Rx moves to the left side of the road, (**d**) Tx on the left (Tx Point number 9), when Rx moves to the right side of the road by the wall.

Figure 15 shows the results of position estimation using the measured RSSI values, indicating the error of the distance between the estimated point and the actual point of Tx, and the difference between the RSSI value measured at that time and simulation. However, considering TX may not be able to receive depending on the number of steps of each TX and RX, it was further evaluated. Figure 15a,b show the average error of the distance when Rx moves on the left side of the road, which is 5.3 m, and when Rx moves in the center of the road, which is 6.8 m. As shown in Figure 15c, the error average when Rx is moved on the right side of the road is 5.0 m, and 5.7 m as a whole. Table 1 shows the error results when the direction of Tx is right and left, as seen from Rx. The error average of the distance is small when TX is on the road on the left side when viewed from Rx, as shown in Figure 16a. This is because the left side is small, as seen from the average error of RSSI. If TX is on the right side, as shown in Figure 16b, it is estimated using RSSI MAP, but the disturbance of the outdoor environment and the accuracy (such as that of the parameters) of the calculation model are considered insufficient.

Furthermore, we considered the points that are more problematic, rather than considering the whole simulation. The first case was the absence of clustering by labeling particles, as shown in Figure 16d, and second was the case where an appropriate cluster selection could not be performed for a cluster that was clustered by labeling, as shown in Figure 16c.

**Figure 17:**
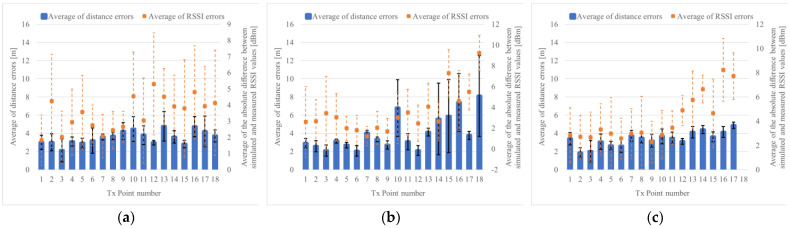
Position estimation results of the actual measurement using the minimum error up to the second ranked cluster in label group selection. (**a**) Result of Rx moves on left side, (**b**) Result of Rx moves on middle side, (**c**) Result of Rx moves on right side.

Regarding the first, there is a problem with clustering even in simulation experiments, which is not peculiar to actual measurement experiments. However, considering it occurs when the distance relationship between Rx and Tx is close, it is not considered a big issue, given that the stranger can be detected for “collision avoidance”. Conversely, regarding the second, when multiple clusters exist by the particle filter, if the cluster selection is correct, the position accuracy (distance accuracy) may be almost correct. Therefore, when selecting a cluster, the minimum error among the errors including the clusters ranked first and second is adopted as the measured value. The results are shown in Figure 17.

The results of the actual measurement experiment show that when Rx moves on the left side of the road, the average error of the distance is 3.2 m, as shown in Figure 17a, and the distance when Rx moves in the center of the road is shown in Figure 17b. When Rx moves on the left side of the road, the error average is 3.3 m, as shown in Figure 17c, the error average of the distance is 3.4 m, and the error average of the distance is 3.3 m, which is a significant decrease. However, although it means that Tx is located in either of the top two positions, it is not known whether it is in one of the corners to realize “collision avoidance”. However, multiple candidates are used as applications. Furthermore, presenting can hinder the contact. Ultimately, if there are multiple candidates, it is sufficient for determining the specific candidate; however, at present, this improvement plan is more practical than unilaterally deciding which one would make a mistake.

Compared with conventional research, the path loss model [4] can measure distances of 3.52 m to 4.47 m and 3 m to 10 m [5], whereas the proposed method can estimate the “position” with the same accuracy. However, with the fingerprint model, although there are studies with the same accuracy [15], the measurement accuracy is within 2 m in an environment with many access points, and machine learning and deep learning [16,17]; therefore, we are considering improving the accuracy through machine learning and deep learning as a future work. Furthermore, the proposed method is different from other methods, as it can measure the position of a stranger using a single receiver.

### 5.2. Actual Measurement Experiment with Tx Moving State

**Figure 18:**
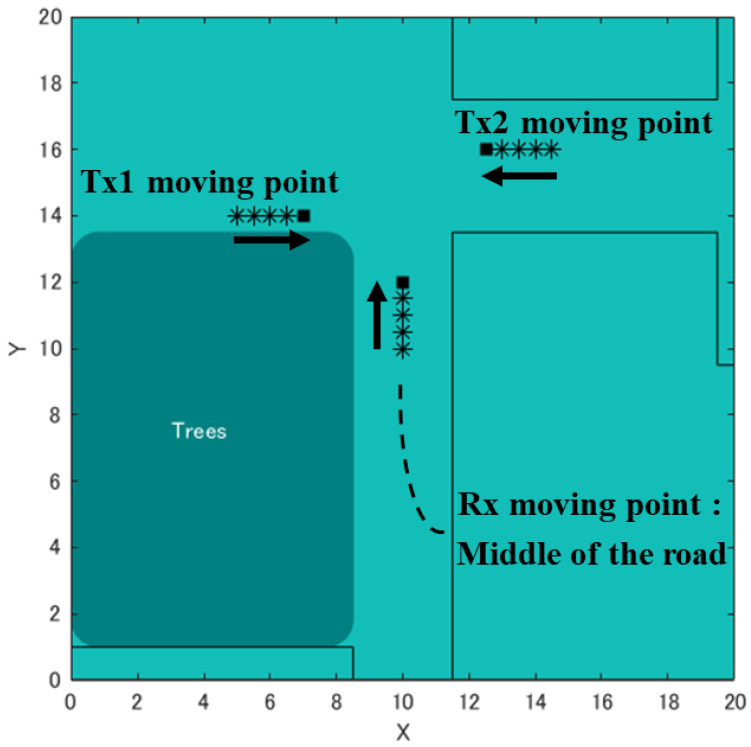
Overview of the point at which Rx and Tx move.

Herein, we perform an evaluation experiment of the proposed method when both Tx and Rx are moving. When two Tx and one Rx are moving, as shown in Figure 18, we determine the positions of the two Tx. Here, Pixel 4a and Pixel 3a are used as Tx, and LINBLE-Z1 is used as Rx. Additionally, we selected a scenario wherein Tx can move and the RSSI value can be obtained from the combination of the position of Tx and the moving area of Rx. It is assumed that Tx1 moves 0.5 m/s from the left and Tx2 moves at the same speed from the right, and the position is estimated from the measurement of each of the five points. This scenario is repeated three times to calculate the average value for further evaluation. Additionally, it is assumed that each person possesses Tx and Rx at a height of 1.2 m in the direction of movement, assuming a person (Tx) walking while looking at the smartphone, and the face and back of the smartphone are the face side and traveling direction side, respectively. Regarding this setting, a study by Douglas et al. [10] reported that if the holding method does not interfere between Rx and Tx, there is almost no human influence on the change in RSSI value depending on the presence or absence of a person. Therefore, situations such as walking with a smartphone can be assumed even for the purpose of “collision prevention”.

**Figure 19:**
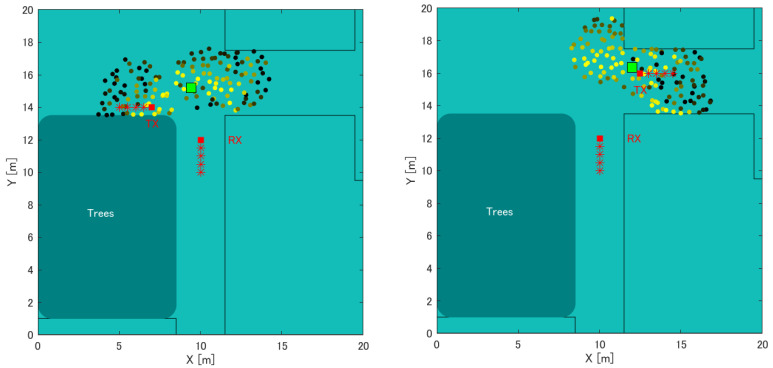
Position estimation results of the measurement simulation in the case of Tx moving. Left: Tx of left direction. Right: Tx of right direction.

**Figure 20:**
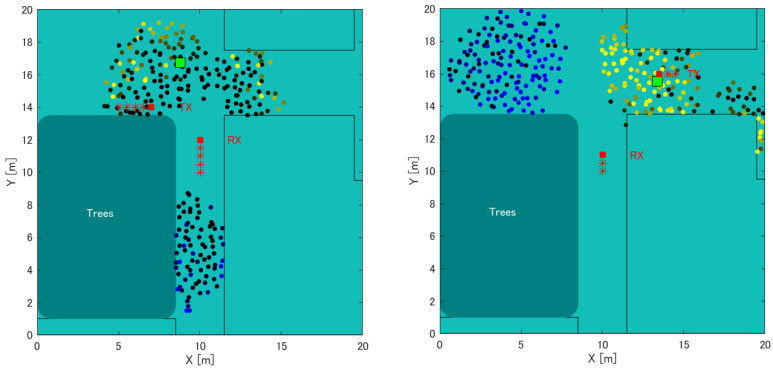
Position estimation results of the actual measurement when Tx is to the left of Rx and moving. Left: Tx of left direction. Right: Tx of right direction.

Table 2. shows the results of the position estimation error using the RSSI value through simulation. Table 3 shows the results of the position estimation error through actual measurement. Figure 19 shows the state of the position estimation of Tx1 and Tx2 as the position estimation using the RSSI value by simulation. Similarly, Figure 20 shows the state of the position estimation of Tx1 and Tx2 as the position estimation using the actually measured RSSI value. As a result, the error average of the distance of the position estimation through simulation and using the actual measured RSSI value was 1.5 m and 4.5 m, respectively. Both Tx1 and Tx2 were less accurate than the average value of 3.3 m for the improvement method in the stationary state. However, the difference was approximately 1 m, which is an acceptable range for the error when moving compared to the stationary state. This confirmed that the proposed method can achieve the same accuracy as when Tx is stationary or when Tx is one, and when Tx is moving or there are two Tx.

## 6. Conclusions

In this study, we proposed a human-to-human position estimation system using BLE RSSI of smartphones to prevent collisions between people at outdoor intersections. Conventional RSSI-based position estimation methods require multiple receivers, which significantly deteriorates the position estimation accuracy, owing to the surrounding buildings. In contrast, in the proposed method, one person carries a single receiver and moves to perform RSSI measurement at multiple points on the trajectory, thereby eliminating the need to install multiple receivers in advance. Additionally, the influence of the surrounding buildings can be considered using the RSSI MAP generated by simulation. Furthermore, in the position estimation algorithm, we proposed a method to apply a particle filter and labeling to narrow down the effective estimated position from multiple candidates. Furthermore, the accuracy of the proposed method was confirmed through simulation in three environmental scenarios. Moreover, we demonstrated the accuracy improvement while using the proposed labeling method. In a T-shaped road simulation model that imitates the actual environment, position estimation simulation and measurement experiments were conducted at 18 Tx points and three Rx movement regions. The results of the simulation and position estimation using the measured RSSI value showed that the error average of the distance was 1.6 m and 5.7 m, respectively. Furthermore, we proposed a method to improve the performance of the system when multiple candidates are present during labeling. The average error was 3.3 m up to the second cluster. Furthermore, we evaluated the accuracy of the proposed method while both Tx and Rx were moving. As a result of position estimation using the measured RSSI value, the average error in distance estimation was 4.5 m. Consequently, we concluded that the accuracy was comparable when the Tx is stationary and when it is moving. Therefore, the proposed method can be used for position acquisition, and it can be expected to be used as a collision avoidance system that can confirm the presence of strangers in the NLOS area.

Future research objectives include improving the clustering part of the particle filter in the position estimation algorithm by using the machine learning and deep learning and real-time generation of RSSIMAP using 3D data of an accurate environmental shape. Since we used LINBLE-Z1 as receiver, we would consider developing a system that considers daily use, using the central function of BLE integrated with a smartphone, because BLE is power saving [18]. To prove the robustness of the proposed method, we will conduct evaluation experiments in environments with complex shapes, such as windows and stairs of buildings, and in places where there are many BLE devices and radio interference occurs. Furthermore, the final map of Tx localization results can be presented to the user as a collision avoidance application. Moreover, recently, research on sensing technology using channel state information (CSI) that can be obtained from Wi-Fi radio waves was conducted [19,20,21]. The main disadvantages of Bluetooth and BLE are that they currently do not provide CSI [22]. However, there is research on measuring BLE tags using methods such as CSI [23]. So, if CSI can be used in our application, we will try to use it.

## Figures and Tables

**Table 1:** Position estimation error results of actual measurement on the T-shape road (direction of Tx as seen from Rx is left: Tx Point 1~9, direction of Tx as seen from Rx is right: Tx Point 10~18).

Moved Area of Rx	Left Side of the Road		Middle of the Road		Right Side of the Road	
Direction of Tx as Seen from Rx	Left	Right	Left	Right	Left	Right
Average of distance errors [m]	4.6	6.1	5.6	8.0	4.3	5.8
Average of RSSI errors [dBm]	2.7	4.1	2.5	4.5	2.8	5.6

**Table 2:** Position estimation error results of the measurement simulation in the case of Tx moving on a T-shape road.

		Direction of Tx as Seen from Rx	
		Left	Right
Distance errors [m]	Average	2.4	0.5
	Standard deviation	0.9	0.3

**Table 3:** Position estimation error results of the actual measurement in the case of Tx moving on a T-shape road.

		Direction of Tx as Seen from Rx	
		Left	Right
Distance errors [m]	Average	4.1	4.8
	Standard deviation	2.9	2.6
Absolute difference between simulated and measured RSSI values [dBm]	Average	2.6	1.5
	Standard deviation	4.1	2.1
